# Exploratory analysis of age and sex dependent DNA methylation patterns on the X-chromosome in whole blood samples

**DOI:** 10.1186/s13073-020-00736-3

**Published:** 2020-04-28

**Authors:** Shuxia Li, Jesper B. Lund, Kaare Christensen, Jan Baumbach, Jonas Mengel-From, Torben Kruse, Weilong Li, Afsaneh Mohammadnejad, Alison Pattie, Riccardo E. Marioni, Ian J. Deary, Qihua Tan

**Affiliations:** 1 Epidemiology and Biostatistics, Department of Public Health, Faculty of Health Science, University of Southern Denmark, J. B. Winsløws Vej 9B, DK-5000 Odense C, Denmark; 2 Unit of Human Genetics, Department of Clinical Research, University of Southern Denmark, Odense, Denmark; 3 Chair of Experimental Bioinformatics, TUM School of Life Sciences Weihenstephan, Technical University of Munich, Freising, Germany; 4 Department of Psychology, University of Edinburgh, Edinburgh, Scotland UK; 5 Centre for Genomic and Experimental Medicine, Institute of Genetics and Molecular Medicine, University of Edinburgh, Edinburgh, Scotland, UK; 6 Lothian Birth Cohorts, University of Edinburgh, Edinburgh, Scotland, UK

**Keywords:** X-chromosome, DNA methylation, X-inactivation, Whole blood, Aging, Twins

## Abstract

**Background:**

Large numbers of autosomal sites are found differentially methylated in the aging genome. Due to analytical difficulties in dealing with sex differences in X-chromosome content and X-inactivation (XCI) in females, this has not been explored for the X chromosome.

**Methods:**

Using data from middle age to elderly individuals (age 55+ years) from two Danish cohorts of monozygotic twins and the Scottish Lothian Birth Cohort 1921, we conducted an X-chromosome-wide analysis of age-associated DNA methylation patterns with consideration of stably inferred XCI status.

**Results:**

Through analysing and comparing sex-specific X-linked DNA methylation changes over age late in life, we identified 123, 293 and 55 CpG sites significant (FDR < 0.05) only in males, only in females and in both sexes of Danish twins. All findings were significantly replicated in the two Danish twin cohorts. CpG sites escaping XCI are predominantly de-methylated with increasing age across cohorts. In contrast, CpGs highly methylated in both sexes are methylated even further with increasing age. Among the replicated sites in Danish samples, 16 (13%), 24 (8.2%) and 3 (5.5%) CpGs were further validated in LBC1921 (FDR < 0.05).

**Conclusions:**

The X-chromosome of whole blood leukocytes displays age- and sex-dependent DNA methylation patterns in relation to XCI across cohorts.

**Electronic supplementary material:**

The online version of this article (10.1186/s13073-020-00736-3) contains supplementary material, which is available to authorized users.

## Background

Aging-related epigenetic changes have been studied intensively using high-throughput techniques for genome-wide DNA methylation profiling. There are reports of large numbers of CpG sites and genomic regions displaying significant age-related methylation changes [1–4]. These age-associated methylation patterns have recently been replicated in independent samples and across populations [5]. Overall, findings from these studies point to extensive epigenetic remodelling in the DNA methylome involving biological pathways related to aging phenotypes and age-related diseases. The X-chromosome comprises about 5% of the human genome and harbours about 800 protein-coding genes; however, age-related methylation changes on this chromosome have typically been ignored. This is probably due to analytical difficulties in dealing with differences in X-chromosome contents of females and males, and X-chromosome inactivation (XCI) in females.

XCI is a unique mechanism of dosage compensation whereby female somatic cells have one X-chromosome randomly repressed, or inactivated, which stems from early embryonic stages in development and throughout subsequent developmental stages. As a result of XCI, one of the X-chromosomes in females has a heterochromatin configuration of the inactive X-chromosome (Xi) whereas the other X-chromosome has euchromatin configuration of the active X-chromosome (Xa). However, approximately 15% of human X-linked genes [6] are also known to escape XCI and are commonly expressed at double dose in females compared to males. After its discovery nearly 60 years ago [7], XCI has been extensively studied especially via mouse models [8]. DNA methylation is an important mechanism in the maintenance of XCI. Recent development in high-throughput genomic analysis enables DNA methylation profiling on the X-chromosomes using microarray and sequencing technologies to characterize the X-linked epigenetic regulation patterns in relation to XCI. For example, by comparing the average levels of X-linked DNA methylation in males with that in females, CpG sites subject to or escaping XCI on the Xi can be consistently inferred across tissues [6, 9]. These approaches have revealed that XCI is accompanied by DNA methylation changes specifically at CpG islands with the biggest increase in methylation occurring at the promoters of genes under XCI [6, 10]. Cotton et al. [6] also analysed the impact of age on X-linked DNA methylation and XCI status reporting no significant correlation. In addition, age-related changes in XCI skewing have been reported but with inconclusive results [11–13].

Using large collections of DNA methylation data on middle-aged and elderly Danish twins from two cohorts and on unrelated individuals from the Scottish-based Lothian Birth Cohort of 1921 (LBC1921) [14, 15], here we first normalized the raw X-chromosome methylation data on male and female samples separately, and not combined as done in previous methods [6, 10]. Then we examined the X-linked DNA methylation levels in the two sexes to characterize the different methylation patterns in relation to XCI in females and analysed the methylation levels as a function of age for different patterns in male and female samples separately. The use of Danish twins helped to control for confounding from unmeasured genetic and rearing environmental factors with enriched statistical power [6, 16]. Discovery and replication were carried out in the two Danish twin cohorts and replicated results were then validated in the Scottish LBC1921 birth cohort.

## Methods

### The middle-aged Danish twins (MADT)

The MADT samples consist of twin pairs born between 1931 and 1952 collected from the Danish Twin Registry [17]. DNA methylation analysis was performed on 492 blood samples (246 twin pairs, 133 male and 113 female pairs) of subjects aged from 55 to 80 years with a mean age of 66 (Table 1). For fair cross-sex comparison of significant findings, we took an equal sample size for both sexes as the minimum sample size of the two sexes in statistical analysis. Because there were more male than female samples, this was done by sequentially taking male samples closest to the mean age of female samples until reaching female sample size. This resulted in 226 male and 226 female twins with an age range of 56–79 years and a mean age of 65 years (Table 1).

**Table 1:** Basic description of samples

	Sample size			Age at blood sampling		Twin pair		
	Male	Female	Total	Range	Mean	MZ	DZ	Total
**Danish**								
MADT all	266	226	492	50–80	66	243	3	246
MADT used*	226	226	452	56–79	65	224	2	226
LSADT all	72	152	224	73–91	79	101	11	112
LSADT used	72	72	144	74–88	79	67	5	72
**Scottish**								
LBC1921 all	286	190	476	78–95	79			
LBC1921 used	190	190	380	78–91	82			

*The subsets were extracted based on their ranked distance to the opposite sex’s mean (i.e. the females of the MADT was taken based on the males of MADT in the order where the ones with ages closest to the male mean age were subtracted first)

### The Longitudinal Study of Aging Danish Twins (LSADT)

The LSADT study, based on the Danish Twin Registry, is a cohort sequential study of elderly Danish twins. LSADT began in 1995 with an assessment of all members of like-sex twin pairs born in Denmark before 1920. Blood samples were drawn during the home visits in 1996 and 1997 from which DNA was isolated and DNA methylation measured recently [18]. Taking equal sample sizes for males and females resulted in the inclusion of 144 individuals (72 each sex) in our analysis with an age range of 74–88 (Table 1). Details on design and data collection were described previously [19].

### The Lothian Birth Cohort 1921 (LBC1921)

The LBC1921 cohort [14, 15] is a sample of community-dwelling, initially relatively healthy older people, all of whom were born in 1921. When first recruited at mean age 79 years, most participants were residing in the Lothian region (Edinburgh and its surrounding areas) of Scotland. We use data on the LBC1921 samples collected in the period 1999–2013 from all participants born in 1921. The initial recruitment included 550 individuals (312 males; 238 females). They have been assessed across five waves. To avoid attrition bias due to loss of participants during follow-up, only data on the most recent wave available from an individual was used, with ages ranging from 78 to 95 years. As shown in Table 1, after fixing equal sample sizes for males and females as was done for MADT, we had 190 male and 190 female samples with an age range of 78–91 and a mean age of 82.

### DNA methylation data

DNA methylation profiles for all samples used in this study were analysed by the same platform, i.e. the Illumina Human Methylation 450K Beadchip (450K array) containing 485,512 CpG sites across the human genome at single nucleotide resolution in genomic DNA from the whole blood. This study only focused on CpGs from the X-chromosome with a total of 11,232 sites. As a comparison, we also analysed chromosome 20, on which 10,383 CpGs (about the same number as the CpGs on X-chromosome) are covered by the 450K array. Experimental details concerning DNA methylation profiling can be found in Svane et al. [18] for Danish twins and in Marioni et al. [20] for the LBC data. The LBC1921 methylation data are accessible through the European Genome-phenome Archive (https://ega-archive.org/studies/EGAS00001000910) with accession number EGAS00001000910 [21]. The LBC1921 data was available as methylation *β* value which is the ratio of intensities between methylated and unmethylated alleles. The *β* values range between 0 and 1 with 0 being unmethylated and 1 fully methylated.

### Array data preprocessing

For each dataset of Danish twins, normalization on the measured X-chromosome DNA methylation levels was performed by subset-quantile within array normalization (SWAN) [22] using the *minfi* R-package, on male and female X-chromosomes separately. Probes with a detection *p* value (a measure of an individual probe’s performance) > 0.01 were treated as missing. CpG sites with more than 5% missing values were removed from the study. In addition, we also removed cross-reactive [23, 24] and polymorphic probes on the X-chromosome leading to a total of 10,096 X-linked CpGs for subsequent analysis. The same procedures for quality control and polymorphic probe filtering were applied to chromosome 20 resulting in 10,379 CpGs for comparative analysis with the X-chromosome. The normalized methylation data was in the form of a *β* value for each site. Details about data preprocessing for the Scottish LBC1921 methylation data can be found in Marioni et al. [20]. Different from the Danish twins for discovery and replication, the methylation data for the LBC1921 validation samples were normalized for males and females jointly.

Before statistical analysis, the methylation *β* values were transformed into M values using the logit transformation with M = log_2_(*β*/(1 − *β*)) for better statistical properties in fitting regression models.

### Correcting for cell type composition

Blood cell-type composition was estimated using Houseman’s methods (based on normalized methylation beta values from 500 differentially methylated regions) for CD8T, CD4T, natural killer cell (NK), B cell, monocyte, and granulocyte using the R packages *minfi* for Danish twins and *celltypes450* (https://github.com/brentp/celltypes450) for the LBC1921s. Correction for cell type composition was done by including the estimated cell type proportions as covariates in the regression models.

### Statistical analysis

#### Permutation test

In order to infer X-inactivation status, we first analysed sex differences in DNA methylation levels by regressing methylation *M*-value on sex (0 for males and 1 for females) in a mixed effect model assigning twin pairing as a random effect variable to account for twin correlation due to shared genetic make-ups. Correction for multiple testing was done by permuting sex for twin pairs to create permuted samples of random sex for *K* = 10,000 replicates. For each replicate, an analysis as per the real data was performed with the lowest *p* value being recorded as Pvalue (perm). The *p* value of each CpG from the real data Pvalue (obs) was compared with the recorded list of Pvalue (perm) to calculate a family-wised error rate (FWER) as

$$ \mathrm{FWER}=\frac{\sum_{i=1}^KI\left[\mathrm{Pvalue}\left(\mathrm{perm}\right)\left(\mathrm{i}\right)<\mathrm{Pvalue}\ \left(\mathrm{obs}\right)\right]}{K}. $$

FWER< 0.05 is used to define sex difference in DNA methylation.

#### Mixed effect modelling

For each X-linked CpG site, we fitted a linear mixed effect model to regress its methylation *M*-value on each participant’s age and cell type proportions with array sentrix barcode and sample sentrix position as random effect variables.

$$ \mathrm{DNAm}={\beta}_0+{\beta}_1\mathrm{age}+{\beta}_2\mathrm{CD}8\mathrm{T}+{\beta}_3\mathrm{CD}4\mathrm{T}+{\beta}_4\mathrm{NK}+{\beta}_5\mathrm{Bcell}+{\beta}_6\mathrm{Mono}+{\beta}_7\mathrm{Gran}+1\left|\mathrm{sentrix}\ \mathrm{ID}+1\right|\mathrm{sentrix}\ \mathrm{position} $$

Age-associated changes in DNA methylation were assessed by examining the regression coefficient for age *β*_*1*_ with *β*_*1*_ > 0 and *β*_*1*_ < 0 indicating an age-associated increase or decrease in DNA methylation. For the Danish twins, the same regression model was fitted but by applying the mixed effect model with an extra random effect variable defined as twin pair ID to account for intra-pair correlation on DNA methylation.

$$ \mathrm{DNAm}={\beta}_0+{\beta}_1\mathrm{age}+{\beta}_2\mathrm{CD}8\mathrm{T}+{\beta}_3\mathrm{CD}4\mathrm{T}+{\beta}_4\mathrm{NK}+{\beta}_5\mathrm{Bcell}+{\beta}_6\mathrm{Mono}+{\beta}_7\mathrm{Gran}+1\mid {\mathrm{TwinPair}}_{\mathrm{ID}}+1\left|\mathrm{sentrix}\ \mathrm{ID}+1\right|\mathrm{sentrix}\ \mathrm{position} $$

The statistical analysis was done for male and female samples separately to avoid sex differences on the X-chromosome while enabling comparison on sex-specific estimates of the slope for age to identify consistent and sex-dependent age-related DNA methylation patterns. Statistical significance of the CpGs was determined by calculating the false discovery rate (FDR) [25] with CpGs with FDR < 0.05 defined as significant.

#### Hypergeometric test

In order to test the significance of the overlaps or replications in the lists of significant CpGs discovered from the two Danish twin cohorts, we applied the hypergeometric test to calculate the probability of randomly observing the number of overlapping or replicated CpGs. The probability of finding *X > k* overlapping CpGs in two lists of significant CpGs from two independent studies can be calculated using the hypergeometric distribution, i.e.


where *N* is the number of CpGs analysed on the X-chromosome, here *N* = 10,096, *m* is the number of significant CpGs from one study, *n* is the number of significant CpGs from another study, and *k* is the number of observed overlapping significant CpGs.

All statistical analysis was performed using R (https://www.r-project.org/) and R-based packages.

## Results

### XCI-related methylation patterns on the X-chromosomes

**Fig. 1:**
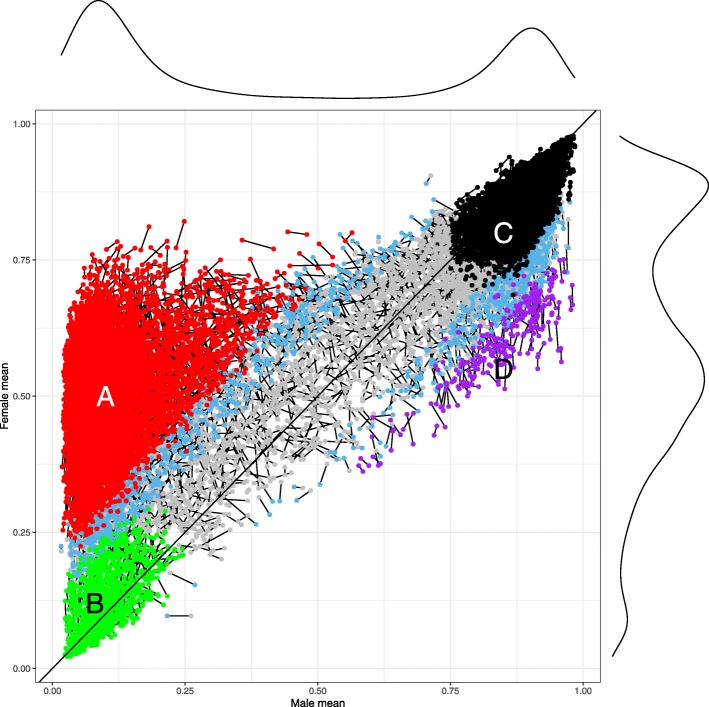
X-linked DNA methylation *β* value in females plotted against that in males for the 10,096 CpG sites revealing sites under XCI significantly more methylated in females than that in males in both LSADT and MADT cohorts coloured red (pattern A), sites escaping XCI with *β* < 0.25 in both sexes with no sex difference (FWER > 0.05) in both cohorts coloured green (pattern B), sites highly methylated with *β* > 0.75 in both sexes with no sex difference (FWER> 0.05) in both cohorts coloured black (pattern C), sites significantly more methylated in males than in females (FWER< 0.05) in both cohorts coloured purple (pattern D) and the rest coloured grey. CpGs coloured light blue are those with sex differential methylation significant only in one cohort. The two dots linked represent the same CpG measured in the two independent cohorts. The curves alongside the *X*- and the *Y*-axis outwards are density curves of *β* values in male and female samples

By normalizing DNA methylation measurements on the X-chromosome in males and females separately, we were able to compare the sex-specific DNA methylation levels at all X-linked CpG sites. In Fig. 1 and Additional file 1: Figure S1, the normalized methylation *β* values (mean of methylation percentage by each sex) are plotted along the X-axis for males and along the Y-axis for females in the two Danish twin cohorts listed in Table 1, MADT (Additional file 1: Figure S1, left) and LSADT (Additional file 1: Figure S1, right). In Fig. 1, the two cohorts are overlaid with the two linked dots representing the same CpG but from two different cohorts. The red dots to the left above the diagonal are CpGs that are significantly more methylated in females than in males (FWER< 0.05) in both cohorts, inferred as subject to XCI (pattern A, 4287 CpGs) (Table 2). The green-coloured dots to the bottom-left are those sites with very low methylation levels (we set *β* < 0.25 as cut-off) and not significant (FWER> 0.05) in either cohorts for either sexes. These CpGs have similarly low methylation levels in both sexes and represent sites that escape XCI in females (pattern B, 648 CpGs). The CpGs shown by black dots on the top-right are similar in both sexes (FWER> 0.05) but are highly methylated (we set *β* > 0.75 as cut-off) across sexes and cohorts (pattern C, 2944 CpGs). Interestingly, we also observe a small group of CpGs (coloured purple) where sites are significantly more methylated in males than in females in both cohorts (FWER< 0.05), indicated as pattern D (149 CpGs). The rest of the sites are either differentially methylated by sex (FWER < 0.05) in the larger MADT cohort only (light blue) or non-differentially methylated by sex (FWER > 0.05) in both cohorts with methylation levels between patterns B and C (light grey) (2068 CpGs). In Additional file 2: Table S1, we provide a list of the 10,069 X-linked CpGs and their methylation patterns grouped into the different colours shown in Additional file 1: Figure S1. Figure 1 presents results for the two cohorts overlaid. The coordinates for the CpGs are close to each other, revealing that their methylation patterns are relatively stable across cohorts. Along the horizontal and vertical axes, we plot the marginal density curves for methylation *β* values in males (top) and females (right). The two sexes exhibit strikingly different patterns characterized by the high density at the two ends of the male curve while in the end and the middle of the female curve due to XCI in females.

**Table 2:** Frequency count of CpGs by gene region for different methylation patterns

**Gene regions**	**Pattern A**	**Pattern B**	**Pattern C**	**Pattern D**	**Rest**	***Total***
**1stExon**	471	43	146	7	123	*790*
**3′UTR**	19	3	281	14	82	*399*
**5′UTR**	711	102	225	38	313	*1389*
**Body**	669	90	891	33	420	*2103*
**Intergenic**	433	114	546	23	388	*1504*
**TSS1500**	854	122	600	24	462	*2062*
**TSS200**	1130	174	255	10	280	*1849*
***Total***	*4287*	*648*	*2944*	*149*	*2068*	*10096*

Chi-squared = 1304.3, df = 24, *p* value < 2.2e−16

### Distribution of XCI-related methylation patterns over gene regions and along the X-chromosome

**Fig. 2:**
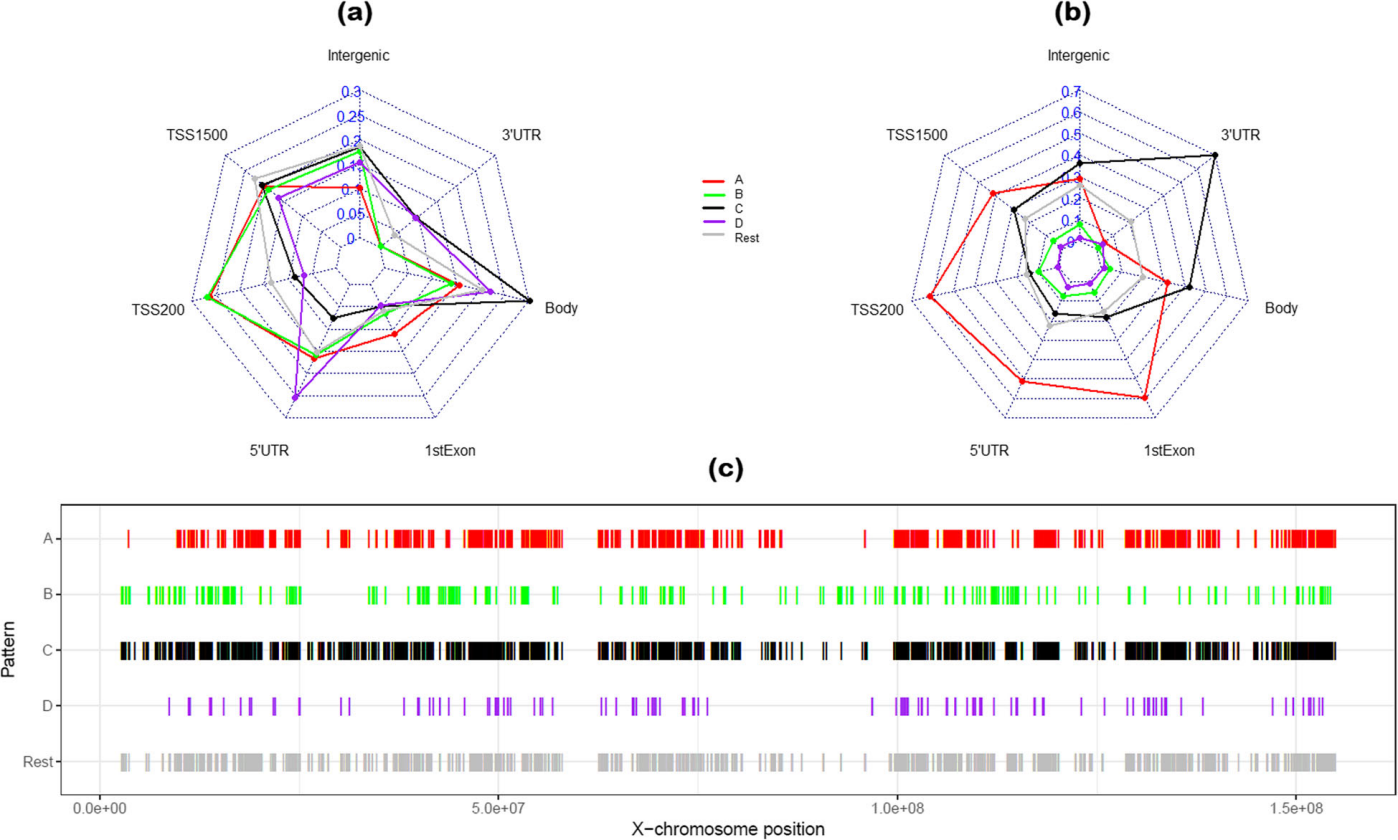
Distribution of methylation patterns in gene regions and along the X-chromosome with **a** a star-plot for proportions of CpGs over gene regions for each pattern (sum for each pattern over regions is 1), **b** a star-plot for proportions of CpGs from different patterns at each region (sum over patterns at each region is 1) and **c** a location map of CpGs form different methylation patterns over the X-chromosome. The colours in the figure represent methylation patterns as in Fig. 1

In Table 2, the number of CpGs coloured for their methylation patterns are distributed according to their locations in gene region (promoter, gene body, intergenic). A chi-squared test showed that the distributions of the different methylation patterns are significantly different over gene regions (*p* < 2.2e-16). This is clearly illustrated by the star-plots in Fig. 2, which displays the proportions of CpGs over gene regions for each methylation pattern (2a) and the proportions of CpGs from different methylation patterns at each gene region (2b). Figure 2a reveals that high proportions of patterns A (subject to XCI) and B (escaping XCI) CpGs are distributed to the promoter region (TSS200, TSS1500, 5′UTR) while pattern C (highly methylated in both sexes) CpGs at gene body. From the absolute frequency in Fig. 2b, the promoter region is overwhelmingly occupied by pattern A CpGs, and 3′UTR is dominated by pattern C CpGs.

In Fig. 2c, we plot the chromosomal locations of CpGs by their patterns on the X-chromosome. The CpGs for patterns A (subject to XCI) and D (male more methylated) show similar distributions along the chromosome while the CpGs in pattern B (escaping XCI) tend to occur more frequently at the lower end of the X-chromosome. Interestingly, the CpGs assigned to pattern C (highly methylated in both sexes) are similarly distributed to the rest of the grey coloured CpGs.

### Age-related changes in X-linked DNA methylation

**Fig. 3:**
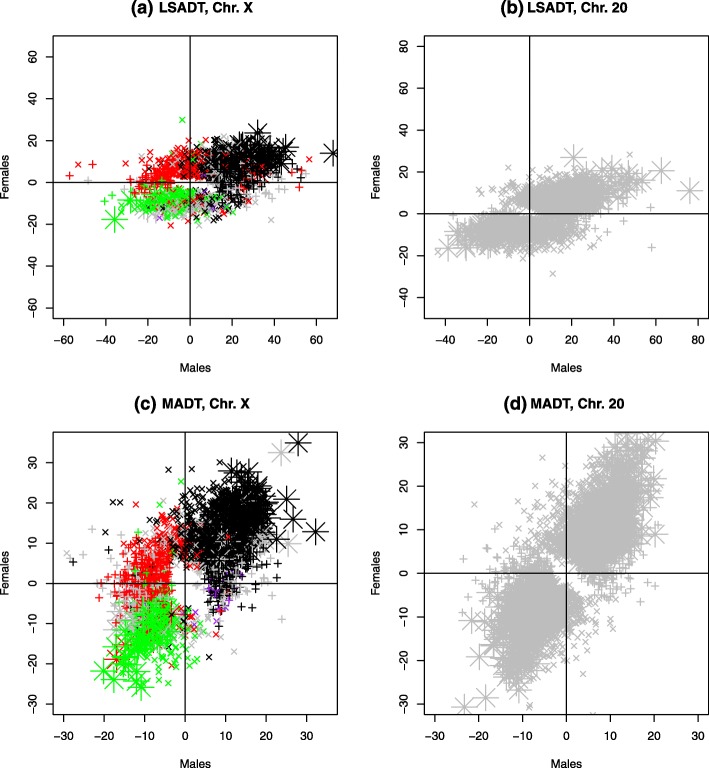
Scatter plots comparing change in DNA methylation by age (regression coefficient of age) of significant CpGs (FDR < 0.05) between male and female samples for sex-specific (plus symbol for male and cross symbol for female) and unspecific (large star symbol) CpGs on the X-chromosome (**a**, **c**) and chromosome 20 (**b**, **d**) in LSADT (**a**, **b**) and MADT (**c**, **d**) cohorts. The colours of CpGs show methylation patterns as in Fig. 1

By applying the linear mixed effect models (with random effect for twin correlation) to the methylation *M*-values in male and female samples separately in the two Danish cohorts (Table 1), we identified X-linked CpGs displaying significant age-associated methylation changes (FDR < 0.05) dependent or independent of sex. The significant sites are dominated by very high proportions of increased methylation (≥ 66%) except for male-only CpGs (i.e. significant only in males) from MADT (34%) (Table 3). In addition, higher proportions of methylated CpGs are observed in the older LSADT cohort than in the relatively younger MADT cohort except for female-only CpGs from LSADT. By plotting the regression coefficient of age (i.e. the rate of change in DNA methylation across ages) in males against that in females, Fig. 3 and Additional file 1: Figure S2 present the methylated CpGs with increasing age that are significant only in males (indicated by a plus symbol), in females (indicated by a cross symbol) or in both sexes (indicated by a star symbol) in the two cohorts respectively. In Fig. 3a for LSADT and 3c for MADT, the X-linked CpGs are coloured similarly as in Fig. 1 to display their pattern-specific age-related methylation changes. Although the figure again reveals the predominant pattern of age-associated methylation increase as in Table 3, it is surprising to see that the methylated CpGs are of pattern C (black) while the de-methylated CpGs are of pattern B (green) CpGs. This is more clearly illustrated in Additional file 1: Figure S2 which plots each pattern individually for LSADT (Figure S2 a-e) and MADT (Figure S2 f-j). Comparing Fig. 3a and c with 3b and d, one can see that, the estimates on chromosome 20 exhibit more consistency between the two sexes, as indicated by the much higher number of sex-independent CpGs on chromosome 20 than that on chromosome X (Table 3). In Table 4, the percentage of significant age-related CpGs (FDR < 0.05) is calculated for each X-linked methylation patterns shown in Fig. 1. In general, the CpGs that are assigned to patterns B and C are similar in both sexes and are more associated with age-related methylation changes. Chi-squared tests show significance in their differential involvement, as explicitly revealed by Additional file 1: Figure S2.

**Table 3:** Number of significant sites (FDR < 0.05) on chromosomes X and 20 by cohort with replication and validation results

	Chromosome X			Chromosome 20		
	Both	Male	Female	Both	Male	Female
LSADT						
Hypermethylated	149	434	897	334	307	1371
Hypomethylated	10	103	462	76	27	2936
% hypermethylated	93.71	80.82	66.00	81.46	91.92	31.83
MADT						
Hypermethylated	325	466	887	734	360	1443
Hypomethylated	90	886	239	749	669	1359
% hypermethylated	78.31	34.47	78.77	49.49	34.99	51.50
LSADT-MADT						
Replicated	55	123	293	204	36	1346
% LSADT	34.59	22.91	21.56	49.76	10.78	31.25
% MADT	13.25	9.10	26.02	13.76	3.50	48.04
Hypergeometric test p	7.89e−46	3.30e−67	3.22e−105	5.42e−157	2.69e−11	0
LBC validation						
Number validated (FDR < 0.05)*	3	16	24	35	15	90
% validated	5.45	13.01	8.19	17.16	41.47	6.69
LBC FDR < 0.05	314	591	2781	611	1180	565

*FDR from testing all CpGs on chromosomes X or 20 in LBC1921 birth cohort

**Table 4:** Age-related CpGs by different methylation patterns

Age association	Pattern A	Pattern B	Pattern C	Pattern D	Rest	*χ*^2^	*p* value
LSADT, female							
FDR > 0.05	3890	463	2269	122	1834		
FDR ≤ 0.05	397	185	675	27	234		
% sig	9.26	28.55	22.93	18.12	11.32	371.62	3.77E−79
MADT, female							
FDR > 0.05	4128	455	1960	137	1875		
FDR ≤ 0.05	159	193	984	12	193		
% sig	3.71	29.78	33.42	8.05	9.33	1361.09	1.89E−293
LSADT, male							
FDR > 0.05	4195	632	2505	140	1928		
FDR ≤ 0.05	92	16	439	9	140		
% sig	2.15	2.47	14.91	6.04	6.77	465.40	2.03E−99
MADT, male							
FDR > 0.05	3664	536	2305	126	1698		
FDR ≤ 0.05	623	112	639	23	370		
% sig	14.53	17.28	21.71	15.44	17.89	62.89	7.17E−13

### Sex-independent age-related methylation patterns

We identified 149 and 325 age-related CpGs significant (FDR < 0.05) in both male and female samples of LSADT and MADT, respectively. Of those, only 10 and 90 CpGs exhibited demethylation with increasing age (Table 3, Additional file 2: Table S2 and Table S3). The proportions of sites with increasing methylation by age were over 78% in both cohorts. Among these sex-independent CpGs, 55 were shared by LSADT and MADT with a hypergeometric probability of 7.89e−46 (Table 3) suggesting the overlap or replication is extremely significant from being random. As shown by Fig. 3 and Additional file 1: Figure S2, the significant age-related and sex-independent CpGs (the stars) are most frequently pattern B (de-methylated), those underlying XCI in females and pattern C (methylated) CpGs, which are de-methylated in both sexes.

### Sex-dependent age-related methylation patterns

Analysis of the male samples found 434 and 466 sites increasing in methylation over time (FDR < 0.05) in LSADT and MADT, respectively (Table 3, Additional file 2: Table S4 and Table S5). There were 103 and 866 CpGs that showed low methylation with age, respectively. A very high proportion of increased methylation (80.82%) was observed in the older LSADT cohort. In comparison, the proportion was only 34.47% in the MADT male samples. Among the male-only significant CpGs, 123 overlapped between LSADT and MADT. The replication is again unlikely due to chance, as highlighted by the very low hypergeometric probability of 3.30e-67.

Analysis of the female samples detected 897 and 887 sites increasing in methylation over time (FDR < 0.05) in LSADT and MADT, respectively (Table 3, Additional file 2: Table S6 and Table S7). There were 462 and 239 CpGs that showed low methylation with age, respectively. The significant CpGs were again mostly methylated with proportions of 66% in LSADT and 78.77% in MADT females. As shown in Table 3, many more age-related significant CpGs were found in female than in male samples within each cohort despite identical sample sizes. Between the two female Danish twin cohorts, 293 significant CpGs overlapped (hypergeometric test *p* value 3.22e−105, Table 3).

### Further validation using LBC1921 birth cohort

The replicated age-associated CpGs from LSADT and MADT cohorts were further validated using the Scottish LBC1921 samples. Of the 471 replicated CpGs in Danish twins, 3 out of the 55 sex-independent CpGs (5.45%), 16 out of 123 male-only (13.01%) and 24 out of 293 female-only (8.19%) CpGs were validated in the LBC1921 samples with FDR < 0.05 (Additional file 2: Table S8). The signs of their coefficients for age showed that they were all methylated with increasing age.

## Discussion

We have performed an exploratory analysis of age-associated DNA methylation changes on the X-chromosome in samples of older people taking account of inferred X-inactivation patterns. By normalizing the raw X-chromosome methylation data in males and females separately and comparing the normalized data across the two sexes, we were able to infer the XCI status and XCI-related DNA methylation patterns using the 450K array data. The fact that the estimated patterns A and B, both groups have low methylation in males, concentrated in the promoter region (Fig. 2) is consistent with previous examinations of XCI from DNA methylation data [6, 26]; thus, we confirm that XCI is readable from DNA methylation data and, in addition, isolating those likely to have escaped XCI (pattern B) form those that undergo XCI (pattern A) (Fig. 1). Note that the CpGs in pattern A are centred around 0.5 on the *Y*-axis. An average methylation percentage below 0.5 in females might suggest incomplete methylation on Xi, perhaps influenced to some extend by X-chromosome reactivation (XCR) [27] as Xi can be prone to partial XCR during development and aging [28, 29].

Previously, Cotton and co-workers concluded no effect of aging on X-linked DNA methylation in a small-scale study on buffy coat samples [6]. By estimating the age-dependent changes (or rate of change in the slope of age in regression) in DNA methylation in male and female samples separately and comparing their differences, we were able to discover the sex-specific and non-specific DNA methylation trajectories across ages. Since our interest is in the slope not the intercept, influence of the complexity in mean level of X-linked methylation is minimized. Moreover, the use of twins in the association analyses helped to control for genetic confounding and to achieve enriched statistical power [16]. As a result, our analysis on monozygotic twins yielded significant age-related methylation changes for CpGs escaping XCI, as well as those under XCI in females. Many of these age-dependent changes were also observed in males.

Very recently, McCartney et al. [30] performed an epigenome-wide association study of sex-specific chronological aging. The study applied linear regression models on sex combined samples and estimated sex-specific effect of DNA methylation (both autosomal and X-linked CpGs) on aging as an interaction between age and sex. Even though the study was on genome scale, their results were overwhelmingly on the X-chromosome (52 autosomal and 597 X-linked CpGs replicated with genome-wide significance defined as 3.6e−08). In order to compare our results with their findings on the X-chromosome, we first mapped their replicated CpGs to our inferred methylation patterns using Danish twins (Fig. 1). In our significant CpGs replicated in Danish twins, the pattern C CpGs have the highest proportion of 59%. In contrast, the X-linked CpGs showing significant sex-specific age patterns in McCartney et al. [30] are predominantly pattern A CpGs (75%). As shown in Fig. 1, the most obvious sex-dependent methylation is characterized by XCI on pattern A CpGs. As a result, a joint analysis of the male and female samples would inevitably favour pattern A CpGs for their sex-specific methylation changes over age. Our analysis normalized the X-chromosome methylation data for males and females separately to avoid influences from sex difference in X-chromosome contents and estimated the sex-specific age patterns again separately in each sex with equal sample sizes so that the estimates are not affected by XCI in females and results on the two sexes comparable. Overall, our analysis shows the necessity in accounting for XCI in handling X-chromosome DNA methylation data both in data analysis and in biological interpretation, as XCI is a unique and highly important epigenetic event occurring on the X-chromosome.

In Fig. 2 a and b, some of the pattern A CpGs are distributed to the gene body. As there have been reports that correlated high DNA methylation level in gene body with increased gene expression [31]. Note that, the CpGs annotated to gene bodies are also frequently annotated to non-transcriptional regions including promoters of other genes. On the other hand, there are also studies reporting negative correlations [32]. The relationship between levels of gene body DNA methylation and gene expression is complex and its link with XCI merits further investigations.

As shown in Table 3 and Fig. 3, the age-related methylation changes are in general characterized by a high proportion of increased methylation. The phenomenon could imply age-related progressive spreading of increased methylation on the X-chromosome in both sexes. This is in contrast to the reported high proportion of age-related demethylation on the autosomal chromosomes [5]. In fact, a high proportion of methylated CpGs with age has also been reported in our recent analysis of the Y-chromosome [33]. These results could mean that the sex chromosomes undergo differential methylation changes during the aging process as compared with that on the autosomal chromosomes. Although we deliberately used the same number of samples for each sex, there were many more significant sites found in female than in male samples of the two twin cohorts (Table 3). Since the male and female sample sizes are equal, the observed difference is more likely due to biological reasons rather than statistical artefacts produced by a power difference resulted from unequal sample sizes. One could postulate that the X-chromosome undergoes more extensive methylation during aging in females than in males. On the other hand, the phenomenon could also occur if the age-associated X-linked DNA methylation profiles in males are more variable than in females. In fact, our recent analysis of age-dependent variability in X-linked CpGs showed significant increase in males but not in females. This is actually also supported by the wider spread of the estimated coefficient for age in males than in females of the older LSADT cohort in Fig. 3a. More research is needed to clarify the issue.

In the bottom of Table 3, the age-associated significant CpGs replicated as overlapping between MADT and LSADT cohorts were further validated by age-related CpGs from LBC1921 (FDR < 0.05). It is interesting to see that the highest validation rate is achieved for replicated CpGs discovered only in males both on X-chromosome and chromosome 20 although the rate is much lower for X-linked CpGs (13%) than the chromosome 20 CpGs (41%). For the replicated CpGs discovered only in females, the validation rate on X-chromosome is slightly higher (8.2%) than on chromosome 20 (6.7%). However, the overall validation rate for the replicated X-chromosome CpGs (9.1%) is about the same as that for the replicated CpGs for chromosome 20 (8.8%), which could indicate that the different normalization scheme applied to LBC1921 data did not seem to specifically affect its performance in validating the replicated CpGs on the X-chromosome.

The age-associated DNA methylation patterns in Fig. 3, Additional file 1: Figure S2 and Table 4 clearly indicate the differential involvement of XCI characterized by increased methylation in pattern C CpGs and decreased methylation in pattern B CpGs with increasing age, a trend observed in both male and female samples of the LSADT and MADT cohorts. The results seem odd because the methylation levels are already low for pattern B CpGs and high for pattern C CpGs. One possible explanation to this could be that the observed methylation levels were already the consequence of continuous demethylation at pattern B CpGs and methylation at pattern C CpGs over the past ages. Considering the highest proportion of pattern C CpGs are from the gene body, the increased methylation with aging could mean that, similar to pattern B CpGs, most of the pattern C CpGs become more active with increasing age. Although the pattern A CpGs are the largest in number, as shown in Table 4, they are least involved in aging. In sum, the patterns B and C CpGs are most involved in age-associated methylation changes in both males and females.

As a comparison, we performed similar analyses on chromosome 20 which has about the same number of CpGs as the X-chromosome measured on the 450K array to ensure similar degree of correction for multiple testing and a direct comparison of the number of significant CpGs identified. In Fig. 3, the scatter plots for the coefficients of age estimated in male and female samples do not show striking differences as compared to the plots for the X-chromosome. As shown in Table 3, more age-related sites were found to be significant only in females than in males both on the X-chromosome and chromosome 20. This could imply that the aging-related significant methylation changes are more frequent in female genomes than in male genomes. As an explanation, we estimated higher variability in autosomal DNA methylation in males than that in females which can lead to reduced power in detecting age-dependent methylation patterns in males. In Table 3, there were more significant sites that are either sex non-specific or only significant in females on chromosome 20 compared to the X-chromosome. This pattern was also observed in the validated CpGs, perhaps an indication that chromosome 20 (here used to represent the autosomal chromosomes) could have been more involved in age-associated methylation changes as compared with the X-chromosome.

The CpGs in Additional file 2: Table S8 are all methylated with increasing age. Among the genes linked to CpGs in Additional file 2: Table S8, *PLXNA3* is annotated with cg15192932 (gene body), one of the 3 sex-independent CpGs shared by MADT and LSADT twin cohorts and replicated in the LBC1921 cohort, all with FDR < 0.05. This gene encodes a class 3 semaphorin receptor and may be involved in cytoskeletal remodelling as well as apoptosis. The gene may be associated with tumour progression and has been found differentially expressed in 15 types of cancer [34]. In Additional file 2: Table S2 and Table S3, cg17662252 located in the body of *GNL3L* gene is methylated with age in both MADT and LSADT cohorts. It has been shown that overexpression of *GNL3L* drives the fraction of genetically defined tumour cells that exhibit markers and tumourigenic properties of tumour initiating cells of enhanced radio-resistance and propensity to metastasize [35]. In view of the observation that methylated CpG in the gene body is accompanied by gene overexpression [36], our estimate on *GNL3L* is consistent with its role in tumour, supporting that some of the age-dependent methylation changes could be implicated in carcinogenesis [37]. This point is further supported by the 16 triple-overlapping male-specific CpGs in Additional file 2: Table S8 which are enriched for “Amplification hot spot 1: colocolized fragile sites and cancer genes in the Xp22.3-p11.1 region” from gene-set enrichment analysis (http://software.broadinstitute.org/gsea/index.jsp). In contrast, no functional pathway is enriched for the 24 triple-overlapping female-specific CpGs in Additional file 2: Table S8.

The reported loss of X-chromosome with age [38], although at a low rate [39], could impact our analysis of age-associated methylation patterns. The loss of X-chromosome, if preferential to Xi [40], would lead to reduced methylation with increasing age, which is contradictory to our detected predominant pattern of age-associated methylation increases. As shown in Fig. 3, the significant CpGs de-methylated with age are mainly CpGs escaping XCI. This could suggest that our detected age-associated methylation patterns do not seem to be affected by the loss of X-chromosome with aging.

The human *XIST* gene is a pseudogene on the X chromosome that acts as a major effector of the X inactivation process. There are 9 CpGs from the 450K array linked to the gene. After quality control, there were 5 and 7 CpGs left in both sexes in LSADT and MADT samples. We plotted the coefficients of age of these CpGs in male and female samples of MADT and LSADT (Additional file 1: Figure S3) with similar colour as in Fig. 1. Interestingly, in the younger MADT cohort, most of the CpGs displayed a similar rate of methylation change with age while in the older LSADT cohort, the age-dependent methylation change is accelerated in males as compared with females. The colours of these CpGs show that they belong to patterns C (4 black colour CpGs from at least one cohort) and D (3 purple colour CpGs from at least one cohort) and 2 grey CpGs (each from one cohort) characterized by higher methylation levels in males than in females or about equally high methylation in both sexes. Compared with the pattern in Additional file 1: Figure S2 c-e for LSADT and h-j for MADT, it is clear that the *XIST*-linked CpGs, like other X-linked CpGs, are more intensively methylated in males than in females in the older LSADT cohort.

Finally, it is necessary to point out that, while the robust inactivation that is occurring across all cell types has been well dissected out as represented in Fig. 1, our data does not allow us to address the issue of cell-type variation in X inactivation process. Particularly, some of the CpGs determined to be escaping XCI may represent within cell-type-specific variable XCI regions. Moreover, the number of X-linked CpG sites covered by the 450K array is only around 1% of the total number of CpGs (about 1.2 million) on the X-chromosome. Considering the limited coverage, interpretation and generalization of our findings in this study should be done with caution. New data including cell-type-specific data collected using high-throughput sequencing techniques such as whole genome bisulfite sequencing should help with replicating and validating our results.

## Conclusions

Our strategic handling of DNA methylation data taking into account of sex difference in X-chromosome content revealed that the X-chromosome of whole blood leukocytes displays age- and sex-dependent DNA methylation patterns in relation to XCI across cohorts.

## Supplementary information

Additional file 1: **Figure S1.** (Cohort-specific X-linked DNA methylation β value in females plotted against that in males of MADT and LSADT). **Figure S2.** (Pattern-specific scatter plots comparing change in DNA methylation by age of significant CpGs between male and female samples). **Figure S3.** (Scatter plots comparing change in DNA methylation by age of CpGs linked to XIST gene between male and female samples).

Additional file 2: **Table S1.** (A list of CpG sites with methylation patterns in Fig. 1 & Additional file 1: Figure S1). **Table S2.** (Sex-independent age-related CpGs from LSADT). **Table S3.** (Sex-independent age-related CpGs from MADT). **Table S4.** (Male-only age-related CpGs from LSADT). **Table S5.** (Male-only age-related CpGs from MADT). **Table S6.** (Female-only age-related CpGs from LSADT). **Table S7.** (Female-only age-related CpGs from MADT). **Table S8.** (LBC validated CpGs).

## Abbreviations

FDR: False discovery rate
FWER: Family-wised error rate
LBC: Lothian Birth Cohort
LSADT: Longitudinal study of aging Danish twins
MADT: Middle-aged Danish twins
SWAN: Subset-quantile within array normalization
XCI: X-chromosome inactivation
XCR: X-chromosome reactivation
Xi: Inactive X-chromosome
Xa: Active X-chromosome
