# Supplementary material for: Exploratory analysis of age and sex dependent DNA methylation patterns on the X-chromosome in whole blood samples
Source: Genome Med. 2020 Apr 28;12:39. doi: 10.1186/s13073-020-00736-3 (PMC7189689; doi:10.1186/s13073-020-00736-3)
Supplement: Supplementary file 1 — Figure S1. (Cohort-specific X-linked DNA methylation β value in females plotted against that in males of MADT and LSADT). Figure S2. (Pattern-specific scatter plots comparing change in DNA methylation by age of significant CpGs between male and female samples). Figure S3. (Scatter plots comparing change in DNA methylation by age of CpGs linked to XIST gene between male and female samples). [file 13073_2020_736_MOESM1_ESM.pdf]

Additional file 1

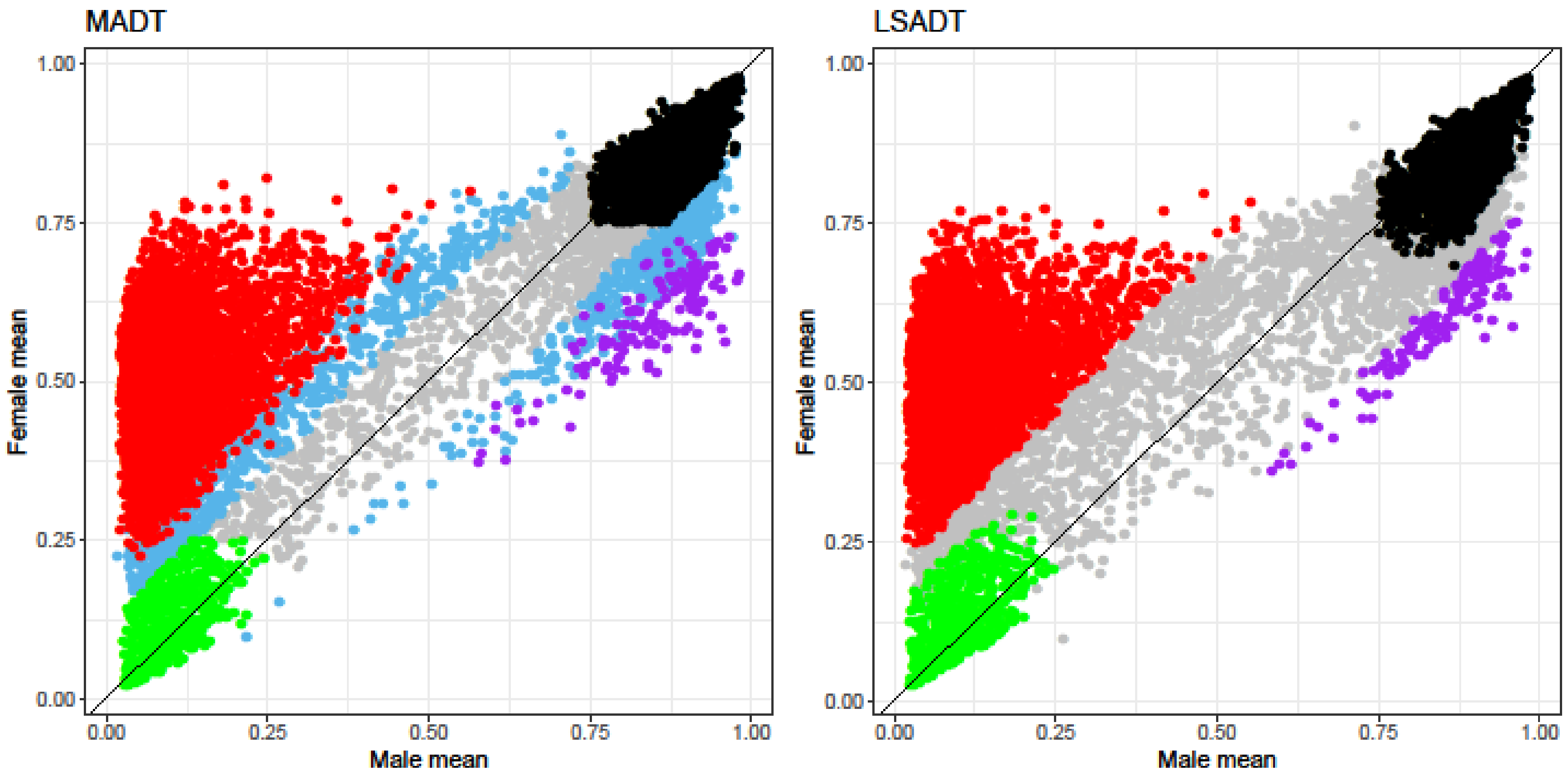

Fig. S1 Cohort-specific X-linked DNA methylation β value in females plotted against that in males for the 10,096 CpG sites revealing sites under XCI, more methylated in females than in males, coloured red (pattern A), sites escaping XCI with β<0.25 in both sexes coloured green (pattern B), sites highly methylated with β>0.75 in both sexes coloured black (pattern C), sites more methylated in males than in females coloured purple (pattern D), sites differentially methylated between sexes but only in MADT coloured light blue, and the rest coloured grey. Patterns A and D are defined by significant differential methylation between the two sexes (FWER<0.05) and patterns B and C are defined by non-differential methylation between the two sexes (FWER>0.05), in both MADT and LSADT cohorts.

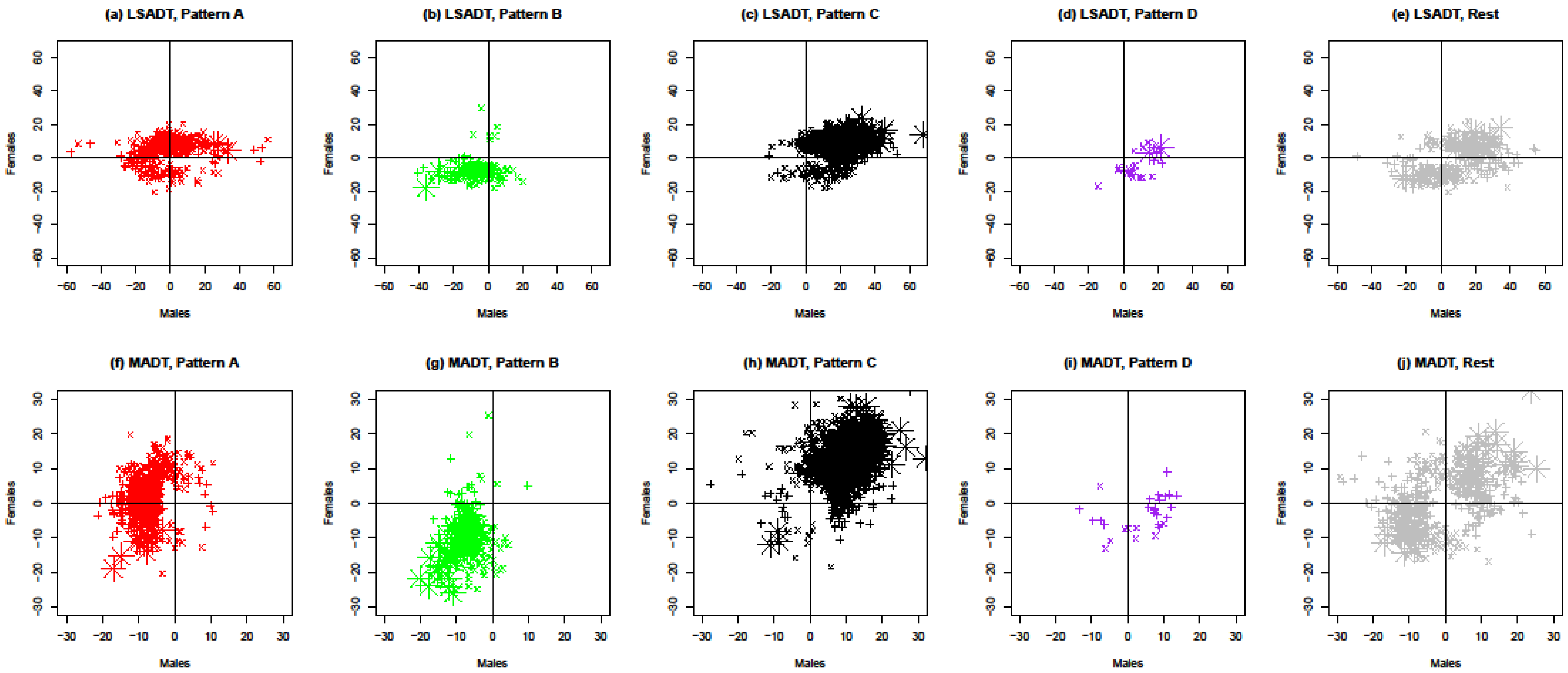

Fig. S2 Scatter plots comparing change in DNA methylation by age (regression coefficient of age) of significant CpGs (FDR<0.05) between male and female samples for sex-specific (plus symbol for male and cross symbol for female) and unspecific (large star symbol) X-linked CpGs plotted for each of the patterns (A-D and the rest) with their corresponding colour as shown in Figure 1 for LSADT (a-e) and MADT (f-j) cohorts.

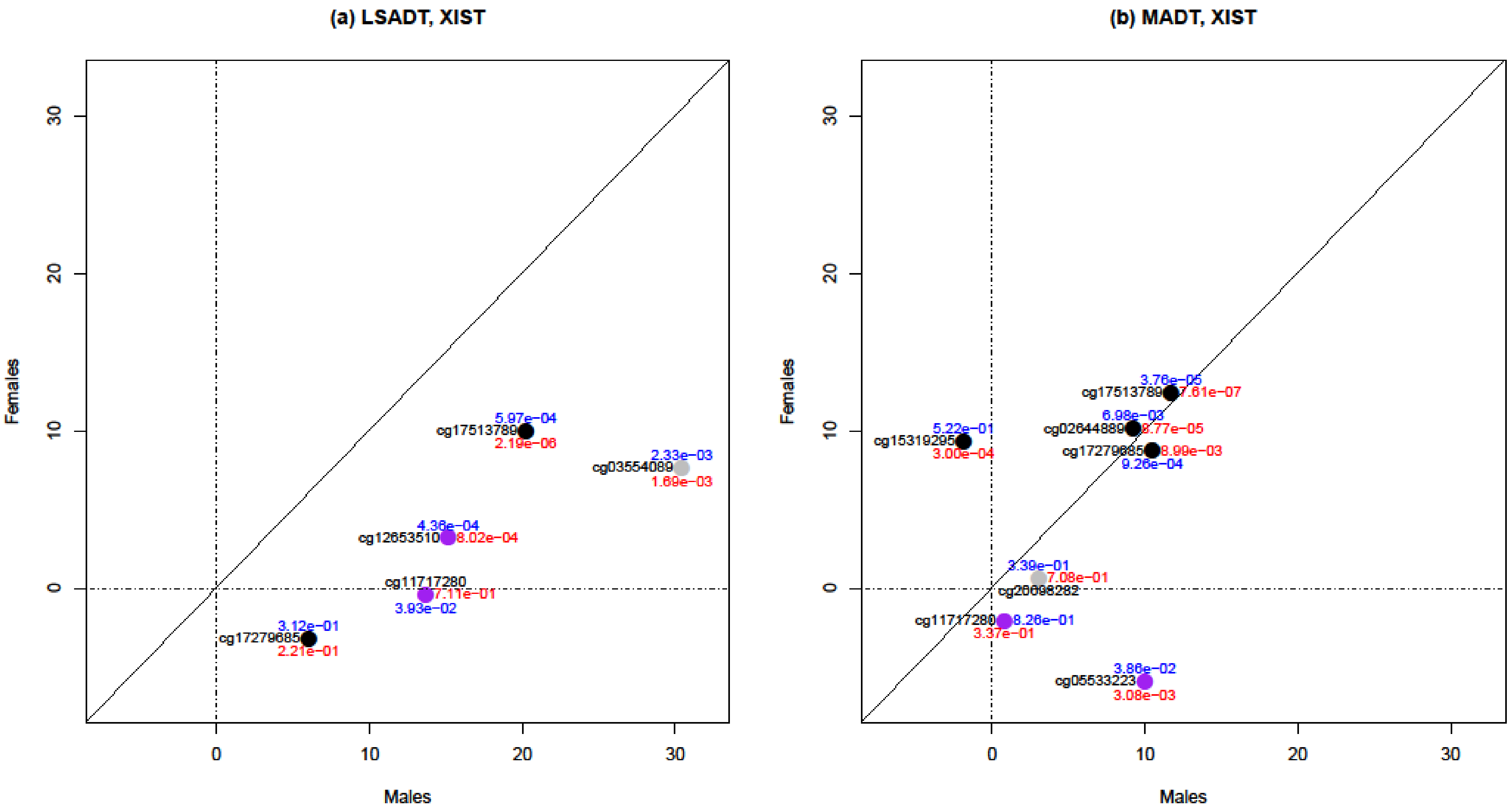

Fig. S3 Scatter plots comparing change in DNA methylation by age (regression coefficient of age) of CpGs linked to XIST gene between male and female samples. The CpGs are annotated by their ID, p-values in males (blue) and in females (red). The colours of CpGs show methylation patterns as in Figure 1.
